# A systematic review and meta-analysis of two different managements for supracondylar humeral fractures in children

**DOI:** 10.1186/s13018-018-0806-1

**Published:** 2018-06-07

**Authors:** Lin-Guo, Xue-Ning Zhang, Jian-Ping Yang, Zhi Wang, Yang Qi, Shan-Zhu, Xiang-Hong Meng

**Affiliations:** 1 Department of Radiology, The Second Hospital of Tianjin Medical University, No 23 PingJiang Road Hexi District, Tianjin, China; 2 Department of Radiology, Tianjin Hospital, No 406 JieFang South Road Hexi District, Tianjin, China; 3 Department of Pediatrics, Tianjin Hospital, Tianjin, China

**Keywords:** Closed reduction, Supracondylar humeral fractures, Meta-analysis

## Abstract

**Background:**

The objective of this meta-analysis was to illustrate the clinical outcomes and safety of two different managements for supracondylar humeral fractures in children.

**Methods:**

In January 2018, a systematic computer-based search was conducted in PubMed, EMBASE, Web of Science, Cochrane Database of Systematic Reviews, and Google database. Data on patients prepared for two different managements for supracondylar humeral fractures in children were retrieved. The primary endpoint was the cosmetic and clinical outcomes based on the criteria of Flynn, ulnar nerve injury, and the occurrence of infection. After testing for publication bias and heterogeneity between studies, data were aggregated for random-effects models when necessary.

**Results:**

Six clinical studies with 581 patients were ultimately included in the meta-analysis. There was no significant difference between the closed reduction and percutaneous cross-pinning, and open reduction and internal fixation in terms of the cosmetic and clinical outcomes based on the criteria of Flynn, ulnar nerve injury, and the occurrence of infection (*P* > 0.05).

**Conclusions:**

Closed reduction and percutaneous pinning, and open reduction and internal fixation of supracondylar humeral fractures in children result in similar construct stability and functional outcome. More high quality randomized controlled trials are needed to identify this conclusion.

**Electronic supplementary material:**

The online version of this article (10.1186/s13018-018-0806-1) contains supplementary material, which is available to authorized users.

## Background

Supracondylar fracture of the humerus is the second most frequent types of bone injury in children [1, 2]. The occurrence of supracondylar fracture of the humerus account for 55 to 75% of patients with elbow fractures [3, 4]. For treatment of this fracture, closed reduction and percutaneous pinning, and open reduction and internal fixation were two common managements for supracondylar fracture of the humerus [5]. Ducic et al. [6] revealed that closed reduction and percutaneous pinning was associated with an increase of the clinical outcomes. Kazimoglu et al. [7] found that closed reduction has equally clinical outcomes than open reduction. Until now, there is no universal agreement among orthopedic surgeons on the most appropriate treatment for supracondylar fracture of the humerus. Currently, there was no relevant meta-analysis that compared closed reduction and open reduction.

Thus, it is necessary to evaluate the efficacy and safety of two different managements for supracondylar humeral fractures in children. This meta-analysis aimed to illustrate the clinical outcomes and safety of two different managements for supracondylar humeral fractures in children. We hypothesize that closed reduction and percutaneous pinning, and open reduction and internal fixation has similar clinical result for supracondylar humeral fractures in children.

## Methods

This systematic review was reported according to the preferred reporting items for systematic reviews and meta-analyses (PRISMA) guidelines [8].

### Search strategies

The following databases were searched in September 2016 without restrictions on location or publication types: PubMed (1950–January 2018), EMBASE (1974–January 2018), the Cochrane Library (January 2018 Issue 3), and the Google database (1950–January 2018). The Mesh terms and their combinations used in the search were as follows: “supracondylar humeral fractures” OR “SCHF” AND “closed reduction” AND “open reduction”. The reference lists of related reviews and original articles were searched for any relevant studies, including RCTs involving adult humans. Only articles originally written in English or translated into English were considered. When multiple reports describing the same sample were published, the most recent or complete report was used. This meta-analysis collected data from published articles and thus no ethic approval was necessary for this article.

### Inclusion criteria and study selection

Patients: patients was diagnose as supracondylar fracture of the humerus surgery; intervention: closed reduction and percutaneous pinning as an intervention group; comparison: open reduction and internal fixation as a comparison group; outcomes: cosmetic and clinical outcomes based on the criteria of Flynn, ulnar nerve injury, and the occurrence of infection; study design: randomized controlled trials (RCTs) and non-RCTs. Two independent reviewers screened the title and abstracts of the identified studies after removing the duplicates from the search results. Any disagreements about the inclusion or exclusion of a study were solved by discussion or consultation with an expert. The reliability of the study selection was determined by Cohen’s kappa test, and the acceptable threshold value was set at 0.61 [6, 7].

### Data abstraction and quality assessment

A specific extraction was conducted to collect data in a pre-generated standard Microsoft® Excel (Microsoft Corporation, Redmond, Washington, USA) file. The items extracted from relevant studies were as follows: first author and publication year, country, sample size of the intervention and control groups, mean age of the intervention and control groups, the protocol of intervention and comparison groups, and follow-ups. Outcomes such as cosmetic and clinical outcomes based on the criteria of Flynn [9], ulnar nerve injury, and the occurrence of infection were abstracted and recorded in the spreadsheet. The criteria of Flynn were presented in Additional file 1. Data in other forms (i.e., median, interquartile range, and mean ± 95% confidence interval (CI)) were converted to the mean ± standard deviation (SD) according to the Cochrane Handbook [10]. If the data were not reported numerically, we extracted these data using the “GetData Graph Digitizer” software from the published figures. All the data were extracted by two independent reviewers, and disagreements were resolved by discussion.

The quality of all included trials was independently assessed by two reviewers on the basis of the Cochrane Handbook for Systematic Reviews of Interventions, version 5.1.0 (http://handbook.cochrane.org) [10]. A total of seven domains were used to assess the overall quality: random sequence generation, allocation concealment, blinding of participant and personnel, blinding of outcome assessment, incomplete outcome data, selective reporting, and other bias. Each domain was measured as low bias, unclear bias, or high bias.

### Outcome measures and statistical analysis

Dichotomous outcomes (cosmetic and clinical outcomes based on the criteria of Flynn [9], ulnar nerve injury, and the occurrence of infection) were expressed as a risk ratio (RR) with 95% CI. Statistical significance was set at *P* < 0.05 to summarize the findings across the trials. Variables in the meta-analysis were calculated using Stata software, version 12.0 (Stata Corp., College Station, TX, USA). Statistical heterogeneity was evaluated using the chi-square test and the I^2^ statistic. When there was no statistical evidence of heterogeneity (*I*^2^ < 50%, *P* > 0.1), a fixed-effects model was adopted; otherwise, a random-effects model was chosen. Publication bias was tested using funnel plots. Publication bias was visually assessed using funnel plots and was quantitatively assessed using Begg’s test.

## Results

### Search results and quality assessment

**Fig. 1:**
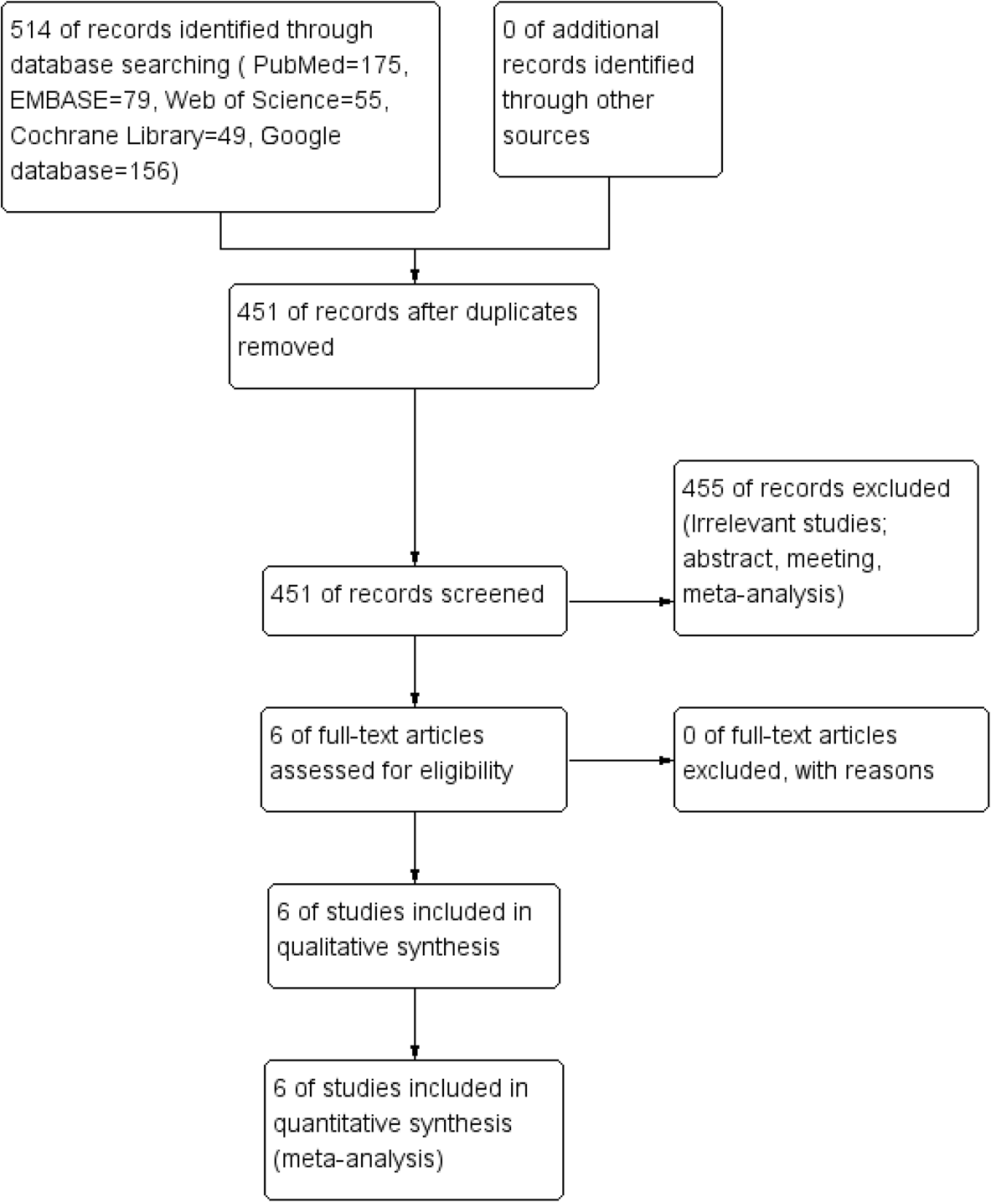
Flowchart of study search and inclusion criteria

Flow of trials through the meta-analysis can be seen in Fig. 1. In the initial search, a total of 514 studies were identified from the electronic databases (PubMed = 175, EMBASE = 79, Web of Science = 55, Cochrane Library = 49, Google database = 156). Then, all papers were input into Endnote X7 (Thomson Reuters Corp., USA) software for the removal of duplicate papers. A total of 451 papers were reviewed, and 455 papers were removed according to the inclusion criteria at abstract and title levels. Finally, a total of six studies were finally included in this meta-analysis [6, 7, 11–14]. The general characteristic of the included studies can be seen in Tables 1 and 2. All of the patients were children, and the mean age ranged from 5.9 to 10.7 years. The type of the fracture mainly focused on the Gartland II and III. The duration of follow-up ranged from 3 to 29.5 months.

**Table 1:** The general characteristic of the included studies

Author	Country	Type of fracture	Age (year)	Intervention	Controls	Outcomes	Follow-up	Study
Ducic 2016 [6]	Serbia	Gartland IIa Gartland IIb Gartland III	6.7 vs 6.1	Closed reduction with percutaneous pinning	Open reduction with Kirschner wire fixation (lateral approach)	1, 2, 3, 4	11.2 ± 2.3 months	RCSs
Kaewpornsawan 2001 [11]	Thailand	NS	7.9 vs 6.8	Closed reduction with percutaneous pinning	Open reduction with Kirschner wire fixation (lateral approach)	1, 2, 3, 4	12 months	RCTs
Kazimoglu 2009 [7]	Turkey	Gartland III	5.9 vs 6.5	Closed reduction and percutaneous cross-pinning	Open reduction and internal fixation (lateral incision)	1, 2, 3	29.5 months	RCSs
Keskin 2014 [12]	Turkey	NS		closed reduction and percutaneous pinning	open reduction and percutaneous pinning(middle incision)	1, 3, 4	3 months	RCSs
Lu 2011 [13]	China	Gartland III	NS	closed reduction and pinning	Open reduction and pinning (middle incision)	1, 2, 3, 4	10 months	RCTs
Ozkoc 2004 [14]	Turkey	Gartland III	10.7 vs 7.6	closed reduction and pinning	Open reduction and pinning (posteromedial incision)	1,2,3,4	21 months	RCSs

*1* cosmetic outcomes based on the criteria of Flynn, *2* clinical outcomes based on the criteria of Flynn, *3* ulnar nerve injury, *4* the occurrence of infection, *PCTs* prospective controlled trials

**Table 2:** The general characteristic of the included studies

Author	Intervention	Controls	Post op protocol	Complications
Ducic 2016 [6]	Two crossed K-wires	ns	K-wires and the cast were removed three to 4 weeks after the procedure	Vascular and neurovascular complications
Kaewpornsawan 2001 [11]	Three pins were inserted laterally by two pins in the upward direction, percutaneously after carefully protecting the ulnar nerve.	Two pins were inserted into upward from the lateral side and one pin downward from the lateral side but the pin did not protrude into the medial condyle	After 4 weeks in a plaster cast, the cast and pins were removed. Bothe groups received same advice concerning a program of physical therapy at home.	Ipsilateral forearm fracture, vascular injury, compartment syndrome, and abnormal growth and development
Kazimoglu 2009 [7]	Eleven patients had been treated with two lateral, 11 patients with one lateral and one medial, and 15 patients with two lateral and one medial K-wires.	Two pins were inserted into upward from the lateral side and one pin downward from the lateral side but the pin did not protrude into the medial condyle	With maximum flexion of 90°	Infection, nerve injury and compartment syndrome
Keskin 2014 [12]	Two Kirschner wires (1.5 mm or 2.0 mm) were placed traversing each other, one from medial and one from lateral aspect for percutaneous fixing while the elbow was locked in full flexion	If the patients having sufficient fracture healing, Kirschner wires were removed without giving anesthesia on an average of 3 weeks after the operation, and active and passive motion of the elbow were allowed	Active elbow range of motion rehabilitation program was encouraged in the fourth week under the supervision of a physical therapist	Vascular and nerve damages, cubitus varus, surgical site and pin tract infection, and other complications
Lu 2011 [13]	Two Kirschner wires (1.5 mm) were placed traversing each other, one from medial and one from lateral aspect for percutaneous fixing while the elbow was locked in full flexion.	ns	The triangle towel suspends the elbow at 90°	ns
Ozkoc 2004 [13]	ns	ns	After the operation three to 4 weeks of dorsal long arm splint at 90°	Compartment syndrome, infection, nerve injuries

*ns*, not stated

### Quality of the included studies

**Fig. 2:**
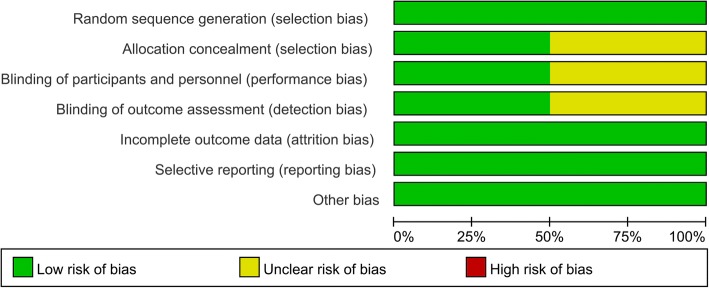
The risk of bias graph

**Fig. 3:**
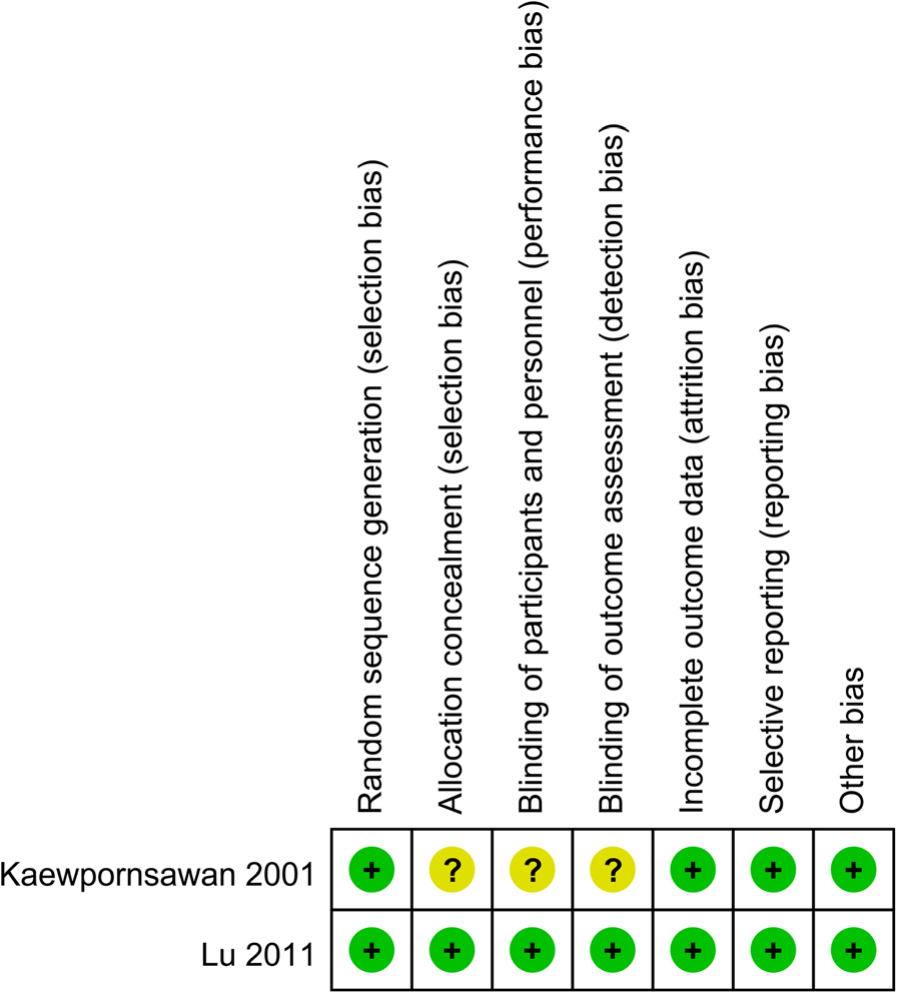
Risk of bias of included randomized controlled trials. +, no bias; −, bias; ?, bias unknown

The quality of RCTs can be seen in Figs. 2 and 3, respectively. Randomized sequence generation was appropriate in one study and the other study was identified as unclear risk of bias. Other selection bias and other bias were unclear risk of bias. The quality of non-RCTs can be seen in Table 3. The scores of the non-RCTs ranged from 16 to 23.

**Table 3:** The Minors quality score of the non-RCTs

First author, year	Minors scale												
	1	2	3	4	5	6	7	8	9	10	11	12	Total
Ducic 2016 [6]	2	1	1	2	0	2	2	0	2	2	1	2	17
Kazimoglu 2009 [7]	2	2	2	2	2	2	2	2	2	1	2	2	23
Keskin 2014 [12]	2	2	0	2	0	2	2	0	2	1	1	2	16
Ozkoc 2004 [14]	2	2	1	2	2	1	2	2	2	2	2	2	22

Numbers 1–12 in heading signified the following: *1* a clearly stated aim, *2* inclusion of consecutive patients, *3* prospective collection of data, *4* endpoints appropriate to the aim of the study, *5* unbiased assessment of the study endpoint, *6* follow-up period appropriate to the aim of the study, *7* loss to follow-up less than 5%, *8* prospective calculation of the study size, *9* an adequate control group, *10* contemporary groups; *11* baseline equivalence of groups, and *12* adequate statistical analyses

### Results of the meta-analysis

#### Functional outcomes based on the criteria of Flynn

**Fig. 4:**
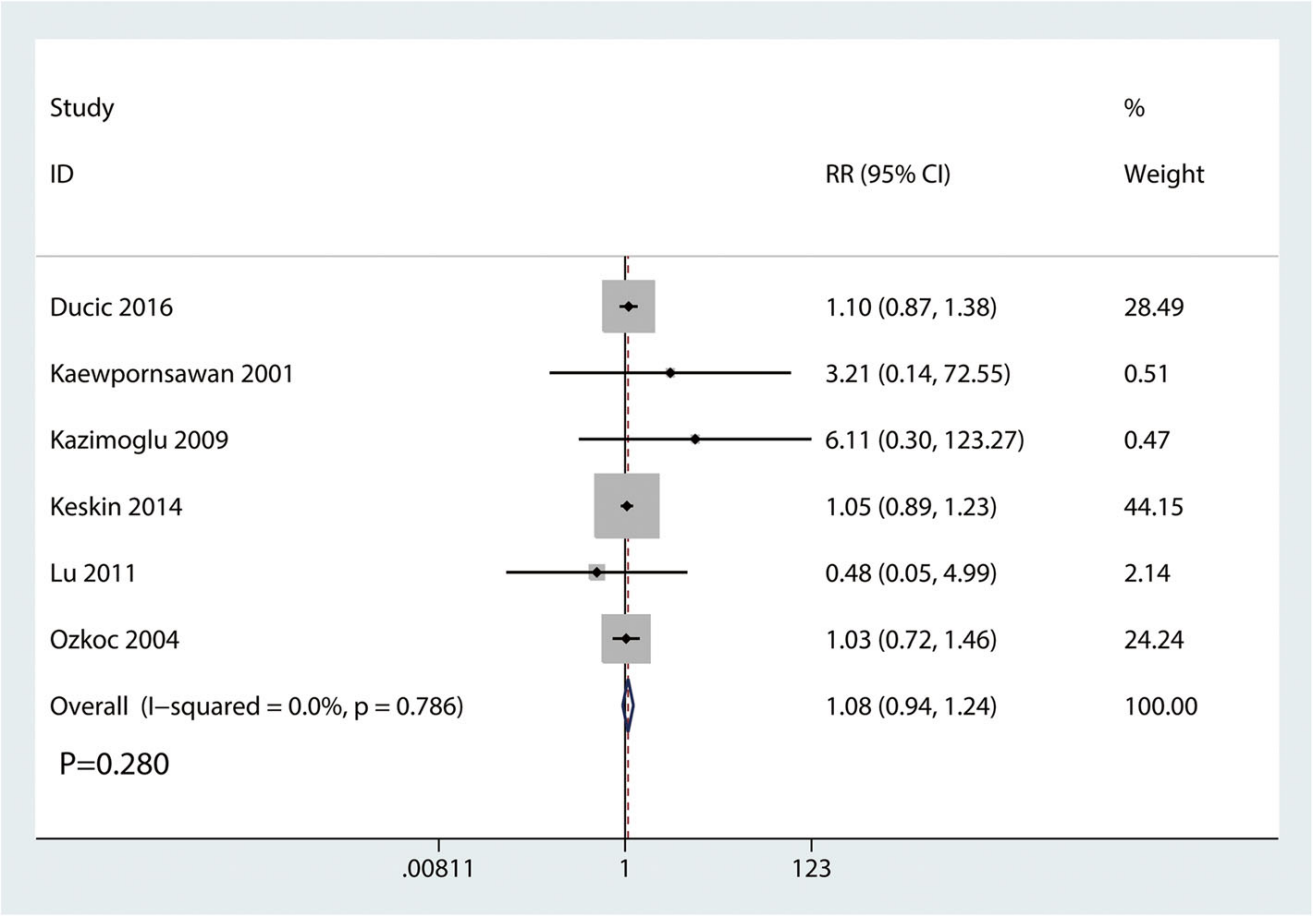
Forest plots of the included studies comparing the cosmetic outcomes based on the criteria of Flynn

**Fig. 5:**
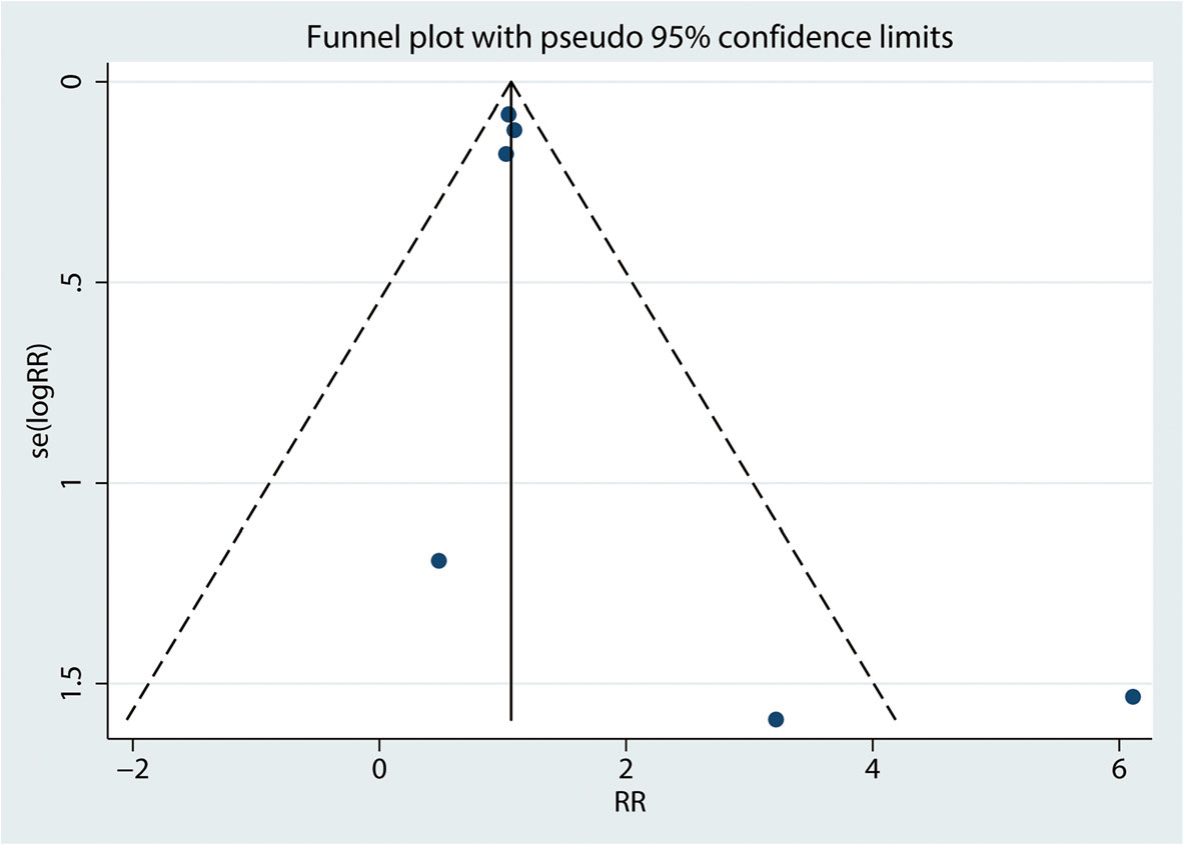
Funnel plot of cosmetic outcomes based on the cosmetic outcomes based on the criteria of Flynn between the two groups

**Fig. 6:**
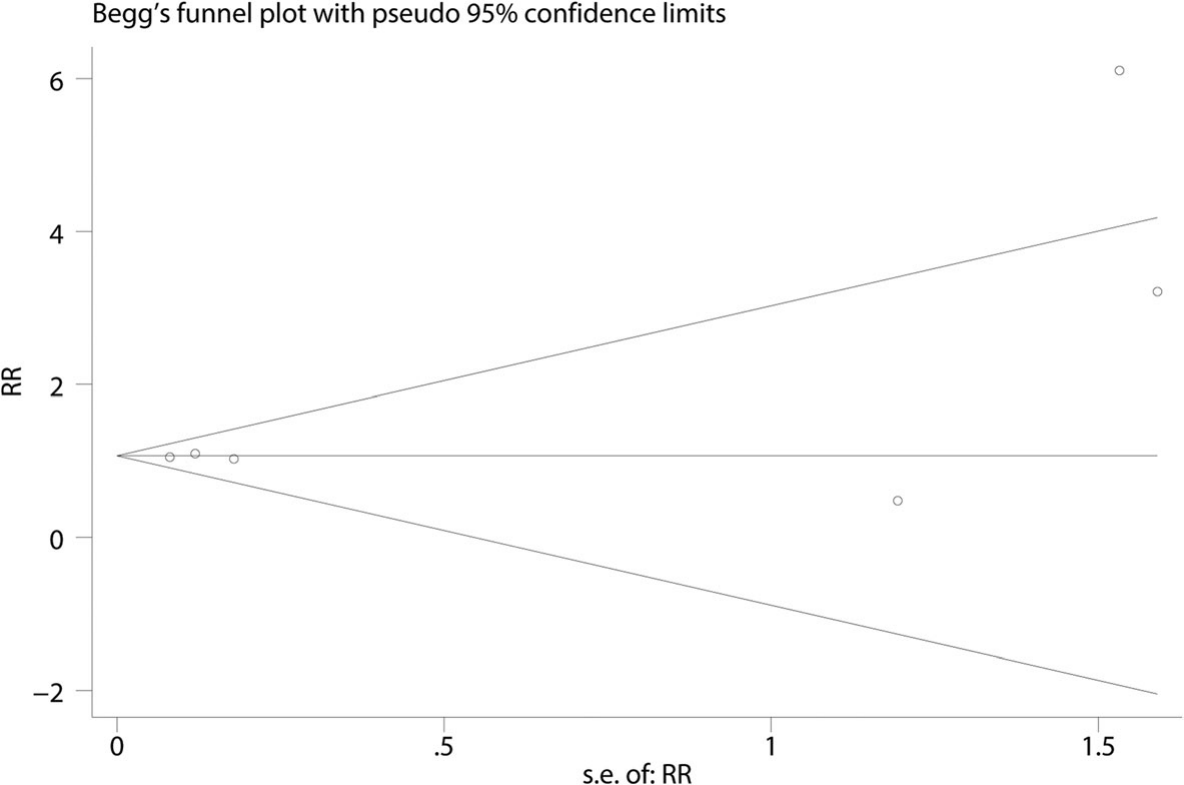
Begg’s test for the functional outcomes based on the criteria of Flynn

**Fig. 7:**
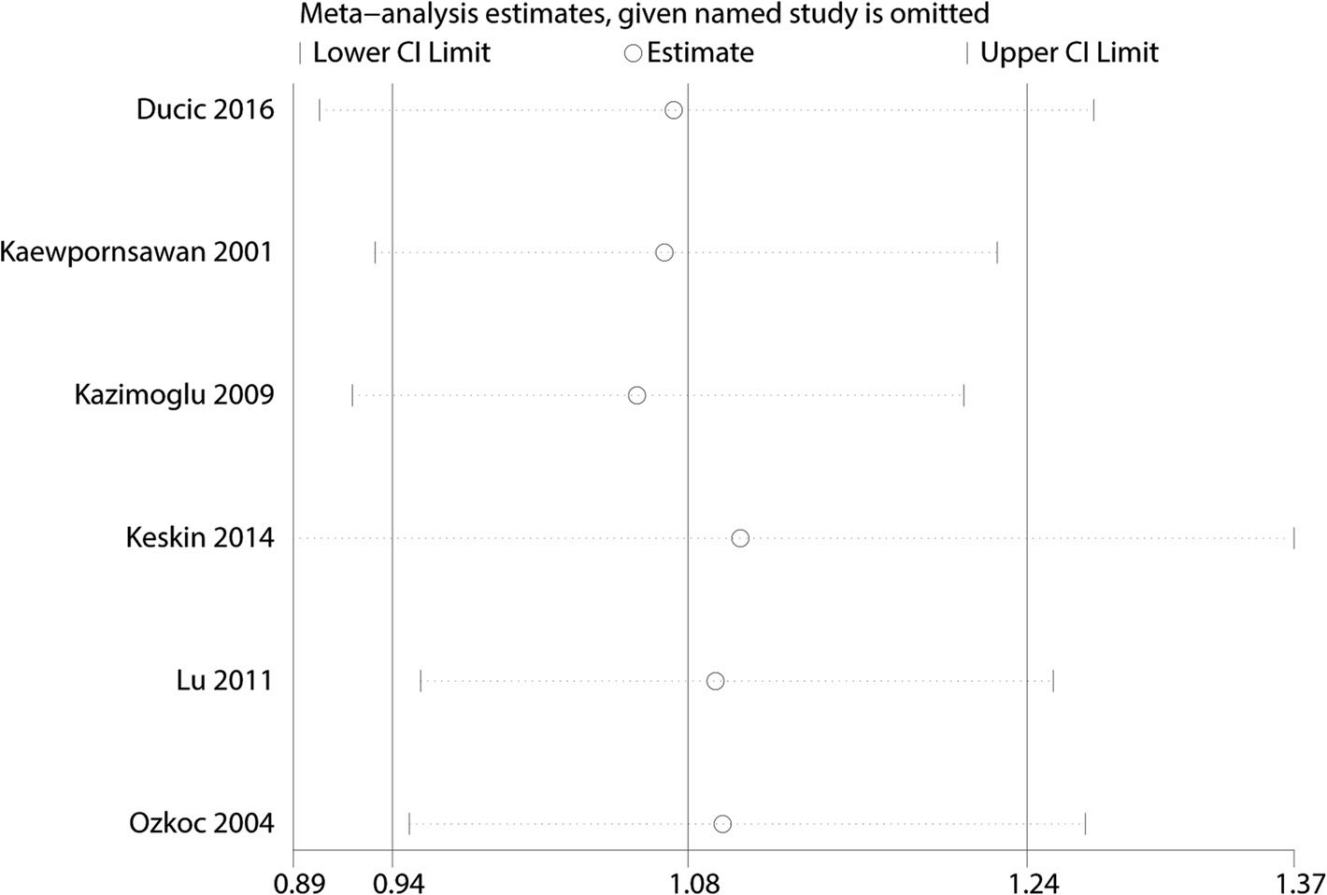
Sensitivity analysis of the functional outcomes based on the criteria of Flynn

Functional outcomes based on the criteria of Flynn were reported in three studies, and the pooled results indicated that there was no significant difference between the cosmetic outcomes based on the criteria of Flynn (RR = 1.08, 95% CI 0.94, 1.24, *P* = 0.280, Fig. 4). Functional outcomes based on the criteria of Flynn has no heterogeneity (*I*^2^ = 0.0%, *P* = 0.786), which required a fixed-effects model that was performed to analyze the data. Funnel plot and Begg’s test were used to identify the potential publication bias of the functional outcomes based on the criteria of Flynn, and results shown that the effect size was symmetrical and there was no publication bias (Figs. 5 and 6). In order to increase the robust of current outcome, sensitivity analysis was performed, and after removal each of the studies, the final outcomes was not changed (Fig. 7).

#### Cosmetic outcomes based on the criteria of Flynn

**Fig. 8:**
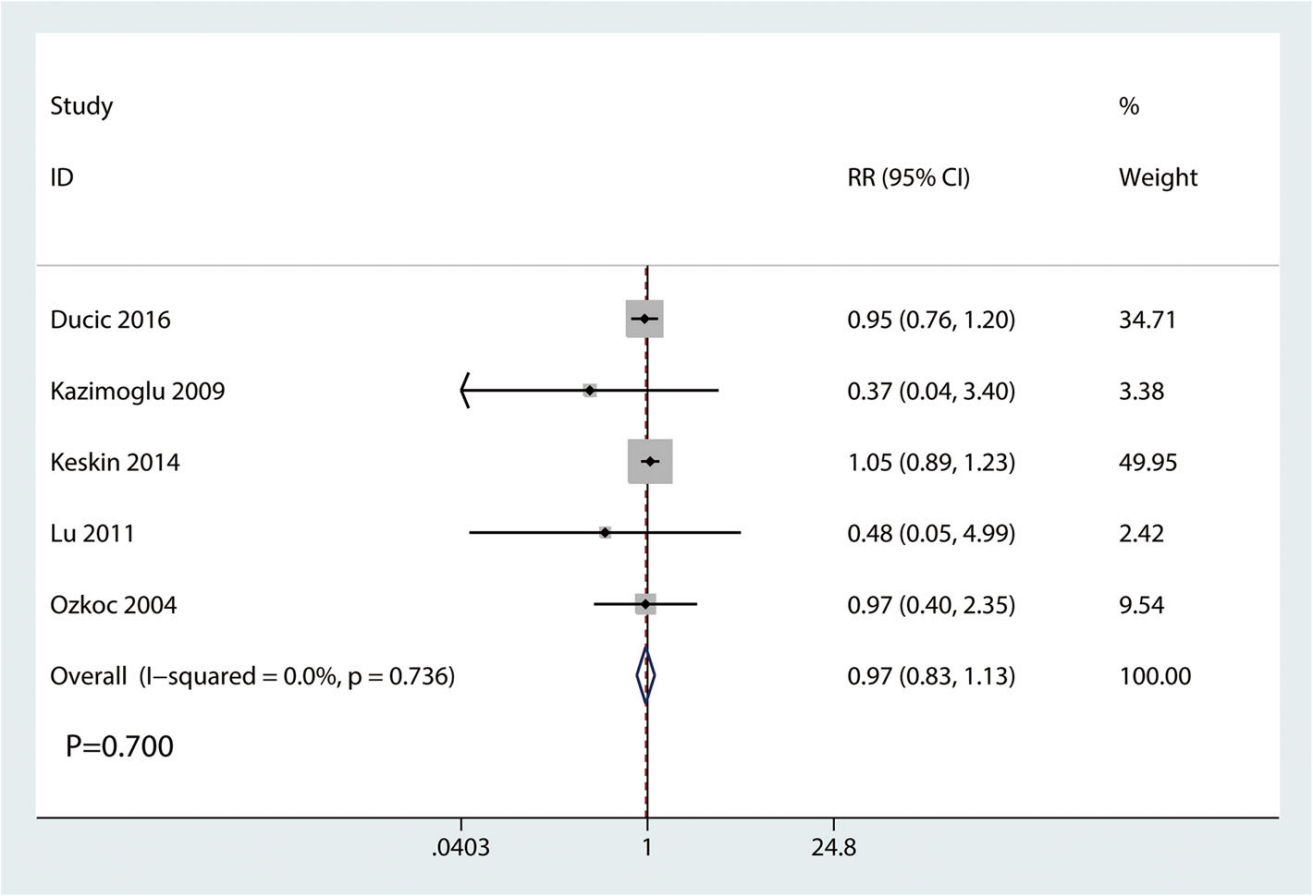
Forest plots of the included studies comparing the cosmetic outcomes based on the criteria of Flynn

Cosmetic outcomes based on the criteria of Flynn were reported in three studies, and the pooled results indicated that there was no significant difference between the cosmetic outcomes based on the criteria of Flynn (RR = 0.97, 95% CI 0.83, 1.13, *P* = 0.700, Fig. 8). Cosmetic outcomes based on the criteria of Flynn has no heterogeneity (*I*^2^ = 0.0%, *P* = 0.736), which required a fixed-effects model that was performed to analyze the data.

### Ulnar nerve injury

**Fig. 9:**
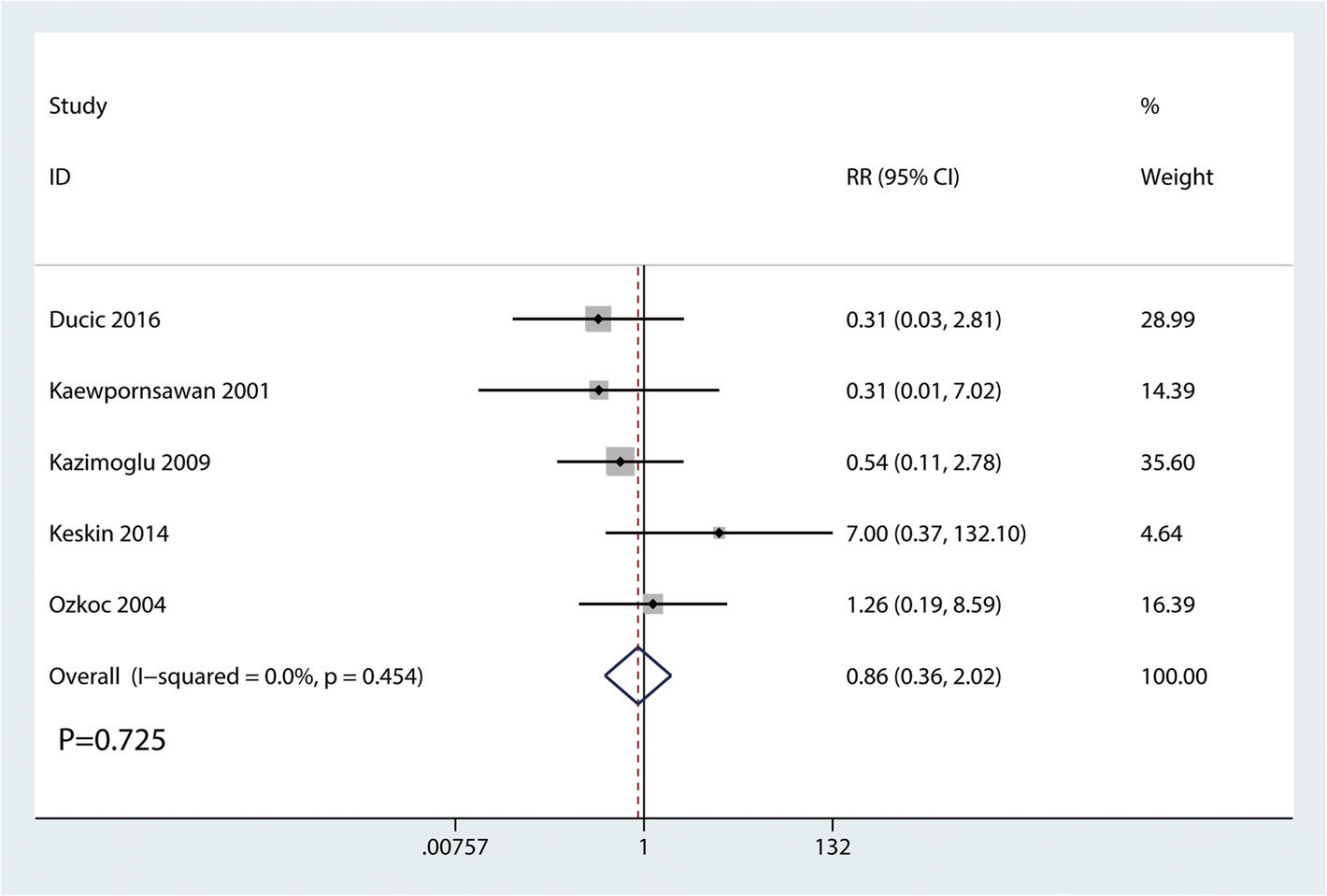
Forest plots of the included studies comparing the occurrence of ulnar nerve injury

Ulnar nerve injury were reported in three studies, and the pooled results indicated that there was no significant difference between the cosmetic outcomes based on the criteria of Flynn (RR = 0.86, 95% CI 0.36, 2.02, *P* = 0.725, Fig. 9). Ulnar nerve injury has no heterogeneity (*I*^2^ = 0.0%, *P* = 0.786), which required a fixed-effects model that was performed to analyze the data.

### Occurrence of infection

**Fig. 10:**
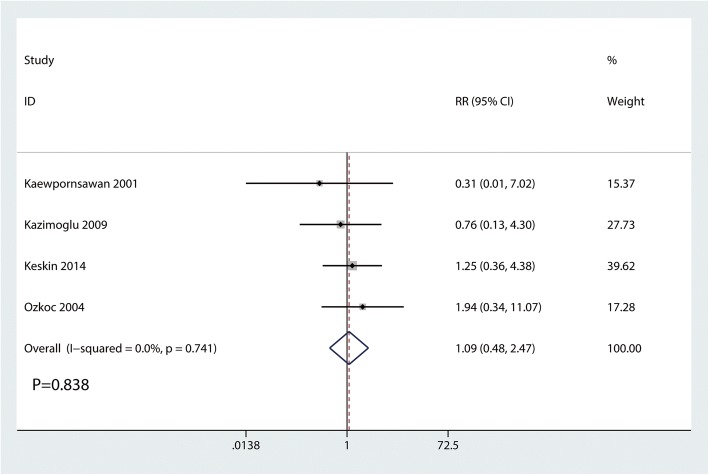
Forest plots of the included studies comparing the occurrence of infection

Occurrence of infection were reported in three studies, and the pooled results indicated that there was no significant difference between the occurrence of infection (RR = 1.09, 95% CI 0.48, 2.47, *P* = 0.838, Fig. 10). Occurrence of infection has no heterogeneity (*I*^2^ = 0.0%, *P* = 0.741), which required a fixed-effects model that was performed to analyze the data.

## Discussion

This is the first systematic review and meta-analysis that comparing different managements for supracondylar humeral fractures in children. Results comprising four outcomes (cosmetic and functional outcomes based on the criteria of Flynn, ulnar nerve injury, and the occurrence of infection). Results shown that there was no significant difference between the above outcomes.

The ideal treatment for supracondylar humeral fractures is, according to many authors, closed reduction and percutaneous pinning. In current study, we found that closed reduction and percutaneous pinning has comparable clinical outcomes according to the criteria of Flynn. Results shown that there was no significant difference between the cosmetic and functional outcomes (*P* > 0.05). Previously, a large number of studies initially tried closed reduction and pinning for supracondylar humeral fractures, and if closed reduction failed and other complications occurred, open reduction was tried. Therefore, open reduction groups generally included the more complicated patients and the clinical outcomes were always bad than closed reduction group. Mulpuri et al. [15] conducted a systematic review and included 44 studies, and they suggested that closed reduction with pin fixation (2 or 3 laterally introduced pins) for patients with displaced supracondylar fractures of the humerus.

And, there was no significant difference between the occurrence of ulnar nerve injury (RR = 0.86, 95% CI 0.36, 2.02, *P* = 0.725). Two lateral pins was an effective and relative stable method to avoid of iatrogenic ulnar nerve injury. Though, cross-pinning was associated with an increase of the occurrence of ulnar nerve injury, long-terms follow-up revealed that ulnar nerve injury will recover spontaneously without complication [16, 17]. Thus, closed reduction was more preferable than open reduction for consideration of the ulnar nerve injury.

The occurrence of infection has been reported as 2.4–6.6% [18–20]. In current meta-analysis, the occurrence of infection for the closed reduction and percutaneous pinning and open reduction was 6.41 and 7.14%, respectively. There was no significant difference between the two groups. These rates were also comparable with reports of previous literatures. Kazimoglu et al. [7] revealed that there was no significant difference between the open group and closed group in terms of the pin tract infection. And all infectious patients were responded well to the oral antibiotic treatment.

There were several limitations in this meta-analysis: (1) only 6 potential studies were finally included, the effect size was relative small; (2) follow-up was relatively short and thus, the potential of these management complications may be underestimated; (3) the management of the K-wires was different and thus, may cause the heterogeneity for the outcomes; (4) subgroup analysis was not performed since the number of the included studies was limited and thus, more RCTs were need to further identify the clinical outcomes of these two managements.

## Conclusion

In conclusion, closed reduction and percutaneous pinning, and open reduction and internal fixation of supracondylar humeral fractures in children result in similar construct stability and functional outcome. And there was no significant difference between the two managements as for the complications. Because the sample size and the number of included studies were limited, a multi-center RCT is needed to identify the effects of closed reduction and percutaneous pinning for supracondylar humeral fractures in children.

## Additional file

Additional file 1: **Table S1.** Flynn Criteria for Grading Supracondylar Humerus Fractures. (DOCX 15.4 kb)

## Abbreviations

CI: Confidence interval
PRISMA: Preferred reporting items for systematic reviews and meta-analyses
RCTs: Randomized controlled trials
RR: Relative risk
SD: Standard deviation
WMD: Weighted mean differences
