# Supplementary material for: A systematic review and meta-analysis of two different managements for supracondylar humeral fractures in children
Source: J Orthop Surg Res. 2018 Jun 7;13:141. doi: 10.1186/s13018-018-0806-1 (PMC5992695; doi:10.1186/s13018-018-0806-1)
Supplement: Supplementary file 1 — Table S1. Flynn Criteria for Grading Supracondylar Humerus Fractures. (DOCX 15.4 kb) [file 13018_2018_806_MOESM1_ESM.docx]

| Results | Rating | Cosmetic Factor: Carrying-angle Loss (deg.) | Functional Factors: Motion Loss (deg.) |
| --- | --- | --- | --- |
| Satisfactory | Excellent | 0-5 | 0-5 |
|  | Good | 5-10 | 5-10 |
| Unsatisfactory | Fair | 10-15 | 10-15 |
|  | Poor | >15 | >15 |

Table 1 Flynn Criteria for Grading Supracondylar Humerus Fractures.
